# An important issue of burnout among pre-hospital emergency medical personnel in Chengdu: a cross-sectional study

**DOI:** 10.1186/s12873-024-00984-1

**Published:** 2024-04-23

**Authors:** ZhiJiang Liu, Li Luo, Hang Dai, Bihua Zhang, Lin Ma, Tao Xiang

**Affiliations:** 1 Chengdu medical emergency center, 610041 Chengdu, China; 2 Department of emergency, The Third People’s Hospital of Chengdu, 610031 Chengdu, China; 3 College of medicine, southwest jiaotong university, 610036 Chengdu, China

**Keywords:** Burnout, Prehospital, Emergency workers

## Abstract

**Objective:**

This survey aims to comprehensively understand occupational burnout among pre-hospital emergency medical personnel and explore associated risk factors.

**Methods:**

A cross-sectional online survey using a census method was conducted between 15 July, 2023, and ends on 14 August, 2023, in Chengdu, SiChuan province, China. The questionnaire included general demographic information, the Maslach Burnout Inventory-General Survey (MBI-GS) with 15 items, and the Fatigue Scale-14 (FS-14) with 14 items. Univariate analysis was conducted on all variables, followed by multivariate logistic regression models to examine the associations between occupational burnout and the risk factors.

**Results:**

A total of 2,299 participants,99.57% completed the survey effectively The participants were from 166 medical institutions in Chengdu, comprising 1,420 nurses (61.50%) and 889 clinical doctors (38.50%). A total of 33.36% participants experienced burnout, predominantly mild (30.27%), followed by moderate (2.78%) and severe (0.3%). Physicians, higher fatigue scores, age, work experience appeared to be related to burnout. Logistic regression models revealed that individuals aged over 50 were less prone to experience burnout compared to medical staff aged 18–30 (OR: 0.269, 95% CI: 0.115–0.627, *p* = 0.002). Physicians were more prone to experience burnout compared to nursing staff (OR: 0.690, 95% CI: 0.531–0.898, *p* = 0.006). Those with 0–5 years of experience were more prone to experience burnout compared to those with 6–10 years or over 15 years of experience (OR: 0.734, 95% CI: 0.547–0.986, *p* = 0.040; OR: 0.559, 95% CI: 0.339–0.924, *p* = 0.023). Additionally, for each 1-point increase in the fatigue score, the likelihood of burnout in medical staff increased by 1.367 times (OR: 1.367, 95% CI: 1.323–1.412, *p* < 0.0001).

**Conclusion:**

Pre-hospital emergency medical personnel demonstrate a notable prevalence of mild job burnout. These results provide a groundwork for future focus on the various stages of job burnout within pre-hospital emergency staff, alerting hospital and departmental managers to promptly address the mental well-being of their personnel and intervene as needed.

Occupational burnout syndrome is a significant issue across various industries, particularly, the medical field, where the demanding nature of the work increases the vulnerability to burnout among emergency department personnel. Burnout has been widely acknowledged as a substantial concern for healthcare professionals, especially those employed in high-stress settings such as emergency departments [1]. The World Health Organization acknowledges that occupational burnout contributes to the deterioration of the health of workers facing difficulties in managing chronic work-related stress [2]. Moreover, studies have demonstrated that work burnout is linked to reduced productivity, elevated employee turnover, an increased absenteeism and turnover intent [3–5]. There is a strong association between the burnout experienced by healthcare workers and significant repercussions, including failed resuscitation attempts and diminished patient care quality [6–9]. Researchers have found a significant correlation among work-related issues, burnout, and job satisfaction, underscoring the importance of addressing burnout-contributing factors within the emergency workers [10].

Pre-hospital emergency care is a crucial component of the emergency department’s responsibilities. It involves the rescue and transportation of injured and ill patients at the scene, as well as their subsequent supervision and transfer to different medical facilities. It takes priority over emergency care in the emergency medical service system and ICU treatment, making it the critical initial step [11]. This work is essential for reducing mortality and disability in emergency patients [12]. A variety of public health emergencies and sudden disaster accidents showed a growing trend, and the prehospital emergency care system has become an important part of the urban security system and public health emergency treatment system [13].The demanding nature of pre-hospital emergency care, characterized by heavy workloads, time-sensitive situations, rapid transitions, and a necessity for high efficiency, demands efficient management and resource allocation in the face of limited resources, uncertainty, lack of recognition, setbacks, and interpersonal conflicts. These factors impose tremendous pressure on pre-hospital emergency care personnel, potentially resulting in occupational burnout. Several studies have shown that job burnout is widespread among prehospital emergency personnel at home and abroad [14–16, 17].. Therefore, the job burnout of prehospital emergency personnel need to attract attention. Gender, age, occupation, education, professional title, years of poerational experience, yeas of work experience in EMS occupation, employment relationship and fatigue were considered risk factors for job burnout among healthcare workers [5, 9, 18, 19]. The current study explores the influencing factors of job burnout among prehospital first responders, the participants were recruited from only one or a few medical institutions. This study specifically targeted medical personnel involved in pre-hospital emergency care in Chengdu, with the objective of examining the prevalence of occupational burnout, the extent of burnout and fatigue, and the factors that contribute to occupational burnout.

## Methods

A cross-sectional study was conducted by wjx(powered by www.wjx.cn) between July 15, 2023, and August 14, 2023 in chengdu, sichuan province, China. The target population of this study was the pre-hospital emergency workers in chengdu. The census method was utilized. This study was approvaled by the Ethics Committee of The Third People’s Hospital of Chengdu (2022-S-108), and completing the questionnaire was considered as informed consent. The documents were distributed to all emergency network hospitals through the Chengdu Health Commission, and participants completed the questionnaire by scanning the quick reponse (QR) code via Wechat. Participants was voluntary and the responses were anonymous. The data was automatically coded and sorted by “Questionnaire Star” to minimize errors introduced by manual input. Lastly, the data was exported to a spreadsheet, stored both in text and digital format. Exclusion criteria for data analysis: (1) incomplete information collection, (2) non-standard information filling, and (3) completion time for the questionnaire less than 180 s.

### Questionnarie and data collection

Before initiating the formal study, we first consulted psychologists working at our institution. The final questionnaire contains general demographic information( gender, age, education level, current position, job title, full-time pre-hospital first aid experience, working unit, hospital grade, total years of work, and years of pre-hospital emergency work.),MBI-GS, and FS-14 with a total of 40 items. Inclusion criteria: All medical personnel currently engaged in pre-hospital emergency care in Chengdu. Exclusion criteria: Previous pre-hospital care providers, retirees, and non-pre-hospital care providers. In Chengdu, the model of pre-hospital emergency care is the simple pre-hospital command type: it is a form of unified dispatch by the pre-hospital emergency center. The pre-hospital emergency personnel in the hospital provide pre-hospital emergency care. These are part-time employees. Only a few hospitals have set up the pre-hospital emergency specialist team, only responsible for the pre-hospital task, which is called full-time staff. However, all of them undertook the same pre-hospital emergency work. We converted some continuous varibles into categorical varibles for the convenience of analysis. We divided working years and years of pre-hospital emergency treatment into four categories: 0-5years, 6-10years, 11-15years, >15years.

### MBI-GS scale

The MBI is widely utilized as a tool for assessing burnout, the most commonly used self-assessment instrument to evaluate the degree of burnout [20–22]. The original MBI has undergone developments and is currently divided into three variations based on the worker’s profession: the Maslach Burnout Scale -Human Services Survey (MBI-HSS), the Maslach Burnout Scale - Educator Survey (MBI-ES), and the Maslach Burnout Scale– General Survey (MBI-GS). The MBI-GS scale is employed across a broad spectrum of occupational groups and comprises three sub-scales: emotional exhaustion, cynicism, and professional efficacy. “Emotional exhaustion” signifies the emotional and physical fatigue resulting from workload and other demands. “Cynicism” denotes a state of indifference or detachment from work, as well as a negative attitude towards work in general. “Professional effectiveness” pertains to the satisfaction with one’s past and present work accomplishments, along with the anticipation of sustained effectiveness in the workplace. The validity of MBI-GS has been confirmed through European studies [23]. MBI-GS has demonstrated reliability and effectiveness across diverse cultural backgrounds and occupations, including healthcare professionals. Prior studies have demonstrated the good reliability, validity, and applicability of the modified version of the General Job Burnout Scale used in China, which comprises 15 items [24]. MBI-GS measures three dimensions of the burnout syndrome: emotional exhaustion (EE; 5 questions), cynicism (DP; 4 questions), and reduced occupational effectiveness (PA; 6 questions). The survey questions revolved around work-related emotions, for example, “I feel that work brings me down.” Respondents expressed the frequency of such sentiments by rating them on a scale from 0 (never) to 6 (daily). Scores should be added and divided by 15 to calculate the average, then multiplied by 20 to obtain a score out of 100. Scores below 50 indicate a favorable working condition, while scores between 50 and 75 suggest a mild burnout. Scores between 75 and 100 indicate moderate burnout, and scores exceeding 100 signify severe burnout. The overall Cronbach’sαcoefficient of the scale was 0.858, indicating that the reliability of the questionaire was good. The KMO test score was 0.925, indicating the structural vlidity of this questionaire was acceptable.

### FS-14

The Fatigue Scale-14 (FS-14) was developed by Trudie Chalder in 1992 as a tool to assess the severity of fatigue symptoms [25]. This scale comprises 14 questions related to fatigue, to which subjects respond either with a yes or no, depending on how well the question aligns with their actual situation. The 14 items cover various aspects of fatigue severity, and can be divided into two main categories: physical fatigue, encompassing items 1 to 8, and mental fatigue, covering items 9 to 14. There are three items, namely 10, 13, and 14, which are scored in reverse, where a no response is given 1 point, while a yes response is given 0 points. The remaining 11 items are scored in the forward direction, with a yes response earning 1 point, and a no response earning 0 points. The overall fatigue score is obtained by summing the scores from both the physical and mental fatigue categories. A higher score indicates a more pronounced level of fatigue. The Cronbach’s α coefficient ranged from 0.88 to 0.90, demonstrating good reliability [14]. Furthermore, the correlation coefficient between individual item scores and the total score ranged from 0.369 to 0.690 (*P* < 0.01), indicating satisfactory validity [25]. In a study involving Chinese doctors and anesthesiologists, the Cronbach’s α coefficient for FS-14 was measured to be 0.844 and 0.73, respectively, suggesting high recognition of the scale within the Chinese medical community and its applicability to all Chinese individuals [26]. The FS-14 not only assesses fatigue severity but also allows for quantitative analysis of the degree of fatigue, making it a widely used scale for quick screening of prolonged fatigue [19].

### Data analysis

Categorical data were analyzed using counts and percentages (%) and compared using either the chi-square or Fisher’s exact test based on appropriateness. The normality of continuous variables was initially assessed using the Shapiro-Wilk test, and if them conformed to a normal distribution, they were presented as mean and standard deviation. Furthermore, the t-test was employed for comparing variables between groups. In cases where variables did not follow a normal distribution, they were described using the median and interquartile range, and compared using the Mann-Whitney U test. A logistic regression analysis was conducted to examine the factors associated with burnout. Variables with an unadjusted P value less than 0.05 were included in the multivariate model, and the results were reported as odds ratios (ORs) with 95% confidence intervals (CIs). Collinearity among the covariates in the final model was assessed by calculating the variance inflation factors(VIF) using linear regression.Collinearity was considered present if the VIF exceed 5. The statistical software used for all analyses was SPSS version 25.0 (IBM Corporation, Armonk, New York, USA). A level of significance was set at *P* < 0.05.

## Results

### The characteristics of participants

Throughout the investigation period, overall, 2309 pre-hospital emergency workers from 166 health institutions in Chengdu participated in the survey. After applying exclusion criteria, 2299 questionnaires were included for statistical analysis, yielding an overall effective rate of 99.57%. Among the participants, there were 1528 females and 771 males, with 39.58% aged 18–30, 41.80% aged 31–40, 15.22% aged 41–50 and 3.39% aged over 50, consisting of 888 doctors (38.63%) and 1411 nurses (61.375%). The personal characteristics are shown in Table 1. Out of the participants, 181 (7.87%) were exclusively involved in pre-hospital first aid, and 2118 (92.13%) were involved in part-time pre-hospital first aid while also performing emergency and first-aid duties in the hospital, 57.76% worked at a Grade A tertiary hospital, 10.35% at a Grade B tertiary hospital, 10.53% at a Grade A secondary hospital, and 6.52% at a Grade B secondary hospital. The ungraded hospital accounted for 14.83%. Regarding education level, 1.70% held a junior college degree, 91.21% held a bachelor’s degree, and 7.09% held a postgraduate degree or higher. Among the respondents’ titles, 51.20% held junior titles, 36.84% held intermediate titles, and 8.74% held senior titles. Additionally, 3.22% were ungraded. Regarding working experience in the medical industry, 24.92% had 0–5 years, 30.01% had 6–10 years, 21.31% had 11–15 years, and 23.75% had over 15 years. Concerning working experience in pre-hospital first aid, 53.55% had 0–5 years, 25.05% had 6–10 years, 14.14% had 11–15 years, and 7.26% had over 15 years. The median fatigue score was 6 (range,3,9). Table 2 presents the relationship between the fatigue score and various factors.

**Table 1:** The characteristics of participants

Variable	N (%)
**Occupation**	
Clinician	888(38.63%)
Nurse	1411(61.37%)
**Gender**	
Male	771(33.54%)
Female	1528(66.46%)
**Age(years)**	
18–30	910(39.58%)
31–40	961(41.80%)
41–50	350(15.22%)
>50	78(3.39%)
**Education**	
Junior college degree	39(1.70%)
Bachelor	2097(91.21%)
Postgraduate	163(7.09%)
**Title**	
Undetermined title	74(3.22%)
Elementary level	1177(51.20%)
Middle level	847(36.84%)
Deputy high official title	169(7.35%)
High level professional title	32(1.39%)
**Years of working**	
0–5	573(24.92%)
6–10	690(30.01%)
11–15	490 (21.31%)
>15	546(23.75%)
**Years of pre-hospital emergency treatment**	
0–5	1231(53.55%)
6–10	576(25.05%)
11–15	325(14.14%)
>15	167(7.26%)
**Full-time staff**	
yes	181(7.87%)
no	2118(92.13%)
**Hospital level**	
Grade A tertiary hospital	1328(57.76%)
Grade B tertiary hospital	238(10.35%)
Grade A secondary hospital	242(10.53%)
Grade B secondary hospital	150(6.52%)
Ungraded hospital	341(14.83%)
**FS-14**	6 (3, 9)

### Proportion of job burnout

**Fig. 1:**
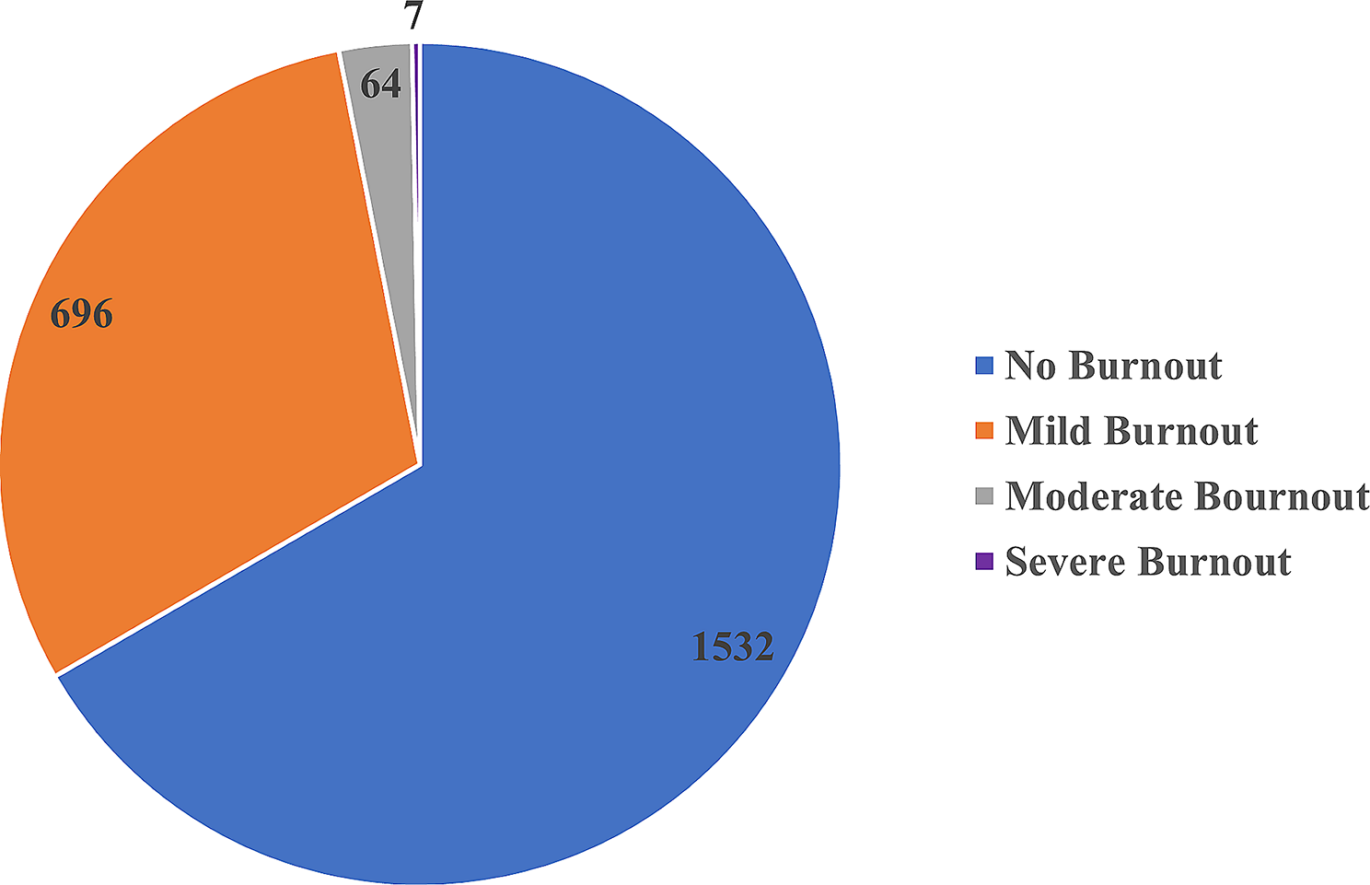
Proportion of job burnout. blue: no bournout; orange: mild bournout; grey: moderate bournout; yellow: severe bournout

Among the 2,299 respondents, 1,532 (66.64%) reported no burnout, 696 (30.27%) reported mild burnout, 64 (2.78%) reported moderate burnout, and 7 (0.3%) reported severe burnout, as displayed in Fig. 1.

### Variables associated with job burnout

Our analysis revealed that position, gender, age, working years, hospital grade, and fatigue were significantly associated with job burnout, as presented in Table 2. Specifically, doctors (328, 36.94%), staff in ungraded hospitals (143, 41.94%) and who with high fatigue scores were more prone to burnout. Conversely, individuals aged over 50 and those with more than 15 years of work experience were less likely to experience job burnout, with statistical significance indicated in Table 2. Although job title and time engaged in pre-hospital first aid did not demonstrate statistical significance in relation to job burnout, we observed a trend indicating that higher job titles and longer engagement in pre-hospital first aid were associated with lower likelihood of experiencing burnout.

The logistic regression model was employed to examine the influence of independent risk factors on job burnout, as depicted in Table 3. After accounting for confounding variables, it was found that healthcare workers aged over 50 were less likely to experience burnout compared to those aged 18–30 (OR: 0.254, 95% CI: 0.109–0.589, *P* = 0.001). Furthermore, medical personnel working in ungraded hospitals were more likely to suffer from burnout when compared to those working in grade A tertiary hospitals (OR: 1.418, 95% CI: 1.075–1.870, *P* = 0.013). While there was no statistical difference observed between workers working in second-class hospitals and those working in third-class hospitals, it is noteworthy that workers working in third-class hospitals exhibited a lower likelihood of experiencing burnout than their counterparts in other hospitals. Moreover, medical staffs who had worked for 0–5 years were more prone to job burnout than those who had worked for 6–10 years or more (OR: 0.741, 95% CI: 0.551–0.996, *P* = 0.047; OR: 0.603, 95% CI: 0.336–0.996, *P* = 0.048). Additionally, an increase of 1 point in the fatigue score corresponded to a 1.369 times greater risk of burnout (OR: 1.369, 95% CI: 1.325–1.413, *P* < 0.0001).

**Table 2:** The univariate analysis the factors associated with job burnout

Variable	Burnout n(%)	Normal n(%)	X^2^/Z	Unadiusted Ρ value
**Occupation**				
Clinician	328(36.94%)	560(63.06%)	8.316	0.004
Nurse	439(31.11%)	972(68.89%)		
**Gender**				
Male	282(36.58%)	489(63.42%)	5.388	0.020
Female	485(31.74%)	1043(68.26%)		
**Age (years)**				
18–30	306(33.63%)	604(66.37%)		
31–40	339(35.28%)	622(64.72%)	13.539	0.004
41–50	110(31.43%)	240(68.57%)		
>50	12(15.38%)	66(84.62%)		
**Education**				
Senior school	10(25.64%)	29(74.36%)		
Bachelor	703(33.52%)	1394(66.48%)	1.075	0.584
Postgraduate	54(33.13%)	109(66.87%)		
**Physician Title**				
Undetermined	27(36.49%)	47(63.51%)		
Elementary	399(33.90%)	778(66.10%)	5.064	0.281
Middle	286(33.77%)	561(66.23%)		
Deputy senior	49(28.99%)	120(71.01%)		
Senior	6(18.75%)	26(81.25%)		
**Years of working**				
0–5	205(35.78%)	368(64.22%)		
6–10	228(33.04%)	462(66.96%)	8.427	0.038
11–15	177 (36.12%)	313(63.88%)		
>15	157(28.75%)	389(71.25%)		
**Years of pre-hospital emergency treatment**				
0–5	416(33.79%)	815(66.21%)		
6–10	206(35.76%)	370(64.24%)		
11–15	95(29.23%)	230(70.77%)	4.972	0.174
>15	50(29.94%)	117(70.06%)		
**Full-time staff**				
yes	69(38.12%)	112(61.88%)	2.002	0.157
no	698(32.96%)	1420(67.04%)		
**Hospital level**				
Grade A tertiary hospital	449(33.81%)	879(66.19%)		
Grade B tertiary hospital	66(27.73%)	172(72.27%)		
Grade A secondary hospital	66(27.27%)	176(72.73%)	20.312	< 0.0001
Grade B secondary hospital	43(28.67%)	107(71.33%)		
Ungraded hospital	143(41.94%)	198(58.06%)		
**FS-14**	9 (6, 11)	5 (3,7)	-20.14	< 0.0001

**Table 3:** The multivariate logistic regression analyses showing the factors associated with job burnout

Variable	OR(95% CI)	Ρ value
**Occupation**		
Clinician	Ref	Ref
Nurse	1.110(0.856-1.439)	0.432
**Gender**		
Male	Ref	Ref
Female	0.813(0.633-1.045)	0.106
**Age(years)**		
18-30 31-40	**Ref** 0.925(0.667-1.281)	**Ref** 0.638
41-50	0.706(0.412-1.208)	0.204
>50	0.254(0.109-0.589)	0.001
**Years of working**		
0-5	**Ref**	**Ref**
6-10	0.741(0.551-0.996)	0.047
11-15	0.750 (0.503-1.120)	0.160
>15	0.603(0.366-0.996)	0.048
**Hospital level**		
Grade A tertiary hospital	**Ref**	**Ref**
Grade B tertiary hospital	0.769(0.545-1.086)	0.136
Grade A secondary hospital	0.830(0.589-1.170)	0.288
GradeB secondary hospital	1.025(0.675-1.556)	0.908
Ungraded hospital	1.418(1.075-1.870)	0.013
**FS-14**	1.369(1.325-1.413)	<0.0001

## Discussion

Burnout is a significant issue among emergency workers, with emergency physicians experiencing a higher burnout rate compared to other specialties [27–30]. In particular, the pre-hospital emergency personnel who have experienced disaster rescue are more prone to post-traumatic stress disorder, bear greater psychological pressure, and are more likely to have job burnout. It has been reported that pre-hospital emergency personnel experience considerable work-related stress, are more prone to various psychological disorders, and have a higher prevalence of post-traumatic stress disorder. Therefore, it is necessary to provide in-service training and psychological intervention for pre-hospital emergency personnel to reduce their stress and increase their psychological resilience after they have completed their tasks [31]. Numerous studies have examined the risk factors and potential consequences of burnout syndrome among emergency workers, particularly in the context of life-threatening illnesses where medical errors and failures can have fatal consequences. The burnout experienced by medical personnel can jeopardize patient safety and health outcomes [32]. However, there is a lack of research on job burnout among pre-hospital emergency workers in China.

Results of studies are controversial where different burnout rates have been reported. Some had reported higher rates of burnout among emergency preofessionals, while others reported lower rates. A survey conducted in Germany in 2018 on 1,101 pre-hospital emergency workers revealed that between 19.9% and 40% of participants experienced high levels of burnout in one dimension [33]. In a study conducted by Dutch researchers in 10 regions, pre-hospital care workers were found to experience fatigue at a rate of 8.6%, which is higher than the 5.3% observed in the general population [33]. Findings from a questionnaire completed by 327 emergency physicians in India indicated a burnout rate of approximately 28.7%, with younger age and lack of respect from colleagues and managers identified as significant factors [34]. EMS professionals perceived high levels of emotional exhaustion and depersonalization(40%), emotional exhaustion(63%), and low level of personal achievement(41.9%) in Riyadh [35]. In a survey conducted with 256 emergency department nurses in six urban areas of Beijing, it was found that 29.7% experienced mild job burnout, 37.5% experienced moderate job burnout, and 31.6% experienced severe job burnout [36]. However, our findings revealed that 30.27% experienced job burnout, and only 3% exhibited severe burnout, necessitating intervention, treatment, and even reassignment in severe cases. Despite the burnout rate in Chengdu being lower than previous records from the Netherlands and Scotland, the prevalence of mild burnout still accounts for approximately one-third of the cases, highlighting the significance of burnout as a noteworthy concern.

The single-factor analysis on job burnout showed a significant relationship between job position and burnout. Specifically, among those experiencing burnout, doctors are more likely to be affected than nurses. Pre-hospital emergency work involves heavy workload, urgent tasks, and a need for high work efficiency. Doctors must make quick diagnosis and treatment decisions while bearing significant pressure and risk throughout the process, leading to a higher burnout rate compared to nursing. These findings align with survey results indicating a high overall incidence of burnout among doctors in China [37]. This finding contrasts with previous surveys and studies on medical personnel, which have generally indicated that nurses are more susceptible to burnout than doctors ^[17.37]^. The differences in the results of these studies may be related to the different institutions and the number of participants. The difference in findings may be attributed to various factors related to in-hospital and pre-hospital work environments. In hospitals, nurses often face challenges such as a high nurse-to-patient ratio, relatively low income, and greater pressure compared to doctors. Nurses experienced moderate levels of depression, stress and anxiety during the COVID-19 pandemic in Iran, and a significant relationship was observed between stressed and gender, level of education and working hours per month [38]. It can be seen that nurses are more prone to mental health problems when they are under greater pressure, which may be caused to the higher burnout scores of nurses. In particular, nurses face greater stress and psychological damage in disasters. The use of telenuring in disasters greatly reduces the post-traumatic stress disorder of nurses on the scene, which is conducive to providing better nursing quality for disasters, reducing the pressure of nursing, and reducing the occurrence of job burnou [39] Telennursing not only provides safe, fast and high-quality care in disaster relief but also during the COVID-19 epidemic, which reduces the need for the number of nursing staff in medical institutions, but also reduces the work intensity of nursing staff [39]. Therefore, this new nursing model can be tried in future management.

Our research indicates that women have a 63.23% higher likelihood of experiencing job burnout compared to men, who have a 36.77% likelihood. A national survey conducted in Iran revealed a high burnout rate among female emergency doctors, with varying levels of burnout across three subscales (84.5%, 48.1%, and 80.5%, respectively) [17]. An analysis of job burnout, involving 606 frontline medical workers recruited from 133 cities during the COVID-19 pandemic, found a significantly higher prevalence of burnout among female employees compared to male employees [18]. In a questionnaire survey involving 1,925 participants, it was found that women were more prone to experiencing job burnout among various first aid professionals [40]. These results are consistent with our findings. Therefore, gender is one of the independent risk factors for burnout. In addition to work, women also shoulder the task of taking care of the family, so they are more likely to suffer from career burnout [38].

The likelihood of job burnout is inversely related to age, overall working years, and professional title. A logistic regression model was utilized to examine the independent risk factors. The results demonstrated that individuals aged over 50 had a lower probability of experiencing job burnout compared to medical professionals aged 18–30. Consistent with our findings, previous studies [17, 41] have consistently revealed that young physicians are more vulnerable to job burnout. Individuals with 0–5 years of work experience are more susceptible to burnout compared to those with 6–10 years or more of experience. Although the level of professional title and the duration of engagement in pre-hospital emergency work did not display statistical significance, individuals with higher professional titles were less likely to experience fatigue. Similarly, a longer duration of engagement in pre-hospital emergency care was associated with a decreased likelihood of burnout. A systematic review and meta-analysis on doctor burnout demonstrated that job title influences job burnout, with individuals holding low job titles experiencing significantly higher levels of emotional exhaustion and burnout compared to those with higher job titles [37]. Likewise, a questionnaire survey involving 1,925 participants illustrated that job burnout was more prevalent among first aid professionals with less than 5 years of work experience compared to professionals with over 10 years of experience [40]. These results align with our findings, indicating an association between low job titles and job burnout. This could be attributed to the insufficient skills and experience of medical staff with junior professional titles. Furthermore, outside the hospital, they often face doubts or lack trust due to their age. Consequently, young medical staff with low professional titles must invest additional time and effort into diagnosis and treatment, despite having less experience in pre-hospital emergency work. They encounter various unexpected situations while still lacking a certain amount of experience, which increases the pressure they bear and heightens the risk of burnout. With the accumulation of work experience, higher professional titles, and increased long-term engagement in pre-hospital work, medical staff can enhance their ability to handle emergency situations and improve their ability to emotionally regulate post-work, leading to a reduction in job burnout.

Fatigue is a significant contributing factor in medical errors and safety hazards. In a study conducted in the United States in 2012, it was found that over half of the participants (55%) from 30 emergency medical services were classified as fatigued [42]. This study also revealed a higher incidence of medical errors and adverse events in fatigued individuals compared to their non-fatigued counterparts. Similarly, our research demonstrated that medical staff with job burnout had significantly higher fatigue scores than those without. By utilizing logistic regression analysis, we identified fatigue as an independent risk factor for burnout, with each 1-point increase in fatigue score increasing the probability risk of burnout by 1.367 times. The issue of work-related fatigue is prevalent among nurses, as evidenced by a survey conducted in Saudi Arabia among emergency department nurses revealing a high degree of nursing fatigue [42]. Additionally, during the COVID-19 period, a survey of 1,794 nurses in Wuhan found that one-third of them experienced fatigue [43]. Hence, it is evident that fatigue is a common problem among medical workers worldwide and warrants attention.

### Limitation

Limitations in the methods of online data collection may have contributed to the reluctance of pre-hospital emergency health care workers to report burnout, which may have contributed to some bias in the results. This study was conducted just chengdu, a city in china. There are three other modes of pre-hospital emergency, the job burnout of pre-hospital emergency personnel may be different. In the future, it is necessary to further analyze the job burnout and its influencing factors under the different pre-hospital emergency mode. Pre-hospital emergency modes: 1. Pre-hospital and in-hospital combination: it is a form of pre-hospital and post-hospital internal and external linkage.2. Simple pre-hospital command type: it is a form of unified dispatch by the pre-hospital emergency center.3. Centralized pre-hospital command type: it is a form of first aid based on the principle of sending the vehicle radius and the cooperation of all parties.4. Pre-hospital affiliated hospital type: it is a kind of pre-hospital command and dispatch is relatively independent, and is affiliated to a general hospital, and all parties are linked at the same time.

## Conclusion

The pre-hospital emergency medical staff exhibit a high prevalence of mild job burnout, while the incidence rate of severe job burnout remains low. age, years of experience, and position were identified as risk factors. This research will enable hospital administrators to identify and address job burnout among pre-hospital emergency medical personnel, leading to improvements in the overall quality of pre-hospital emergency services. In the future study, we plan to increase the influencing factors of job burnout of pre-hospital emergency personnel, including family factors, working hours, demand for pre-hospital emergency care, leave system, hospital and department management system, and this analysis is conducive to hospitals and departments to improve management measures. In addition, mental health investigation and psychological intervention were planned to compare the effect of mental health intervention on job burnout in pre-hospital emergency personnel.

## Data Availability

The original data supporting the conclusions of this paper will be provided by the authors, without reservation. If someone wants to request the data from this study, please contact the correspondence.
